# High cerebrospinal fluid lactate concentration at 48 h of hospital admission predicts poor outcomes in patients with tuberculous meningitis: A multicenter retrospective cohort study

**DOI:** 10.3389/fneur.2022.989832

**Published:** 2022-10-06

**Authors:** Chenchao Liu, Ruixue Huai, Yijia Xiang, Xu Han, Zixiang Chen, Yuhan Liu, Xingjun Liu, Huiquan Liu, Hong Zhang, Sihan Wang, Lingyu Hao, Yin Bo, Yuanbo Luo, Yiyi Wang, Yi Wang

**Affiliations:** ^1^Department of Neurology, Haihe Clinical School, Tianjin Medical University, Tianjin, China; ^2^TCM Key Research Laboratory for Infectious Disease Prevention for State Administration of Traditional Chinese Medicine, Tianjin, China; ^3^Department of Neurosurgery, Tianjin Medical University General Hospital, Tianjin, China; ^4^Key Laboratory of Post-trauma Neuro-Repair and Regeneration in Central Nervous System, Ministry of Education and Key Laboratory of Injuries, Variations and Regeneration of Nervous System, Tianjin Neurological Institute, Tianjin, China; ^5^Department of Neurology, Tianjin Jinnan Hospital, Tianjin, China; ^6^Department of Neurosurgery, People's Liberation Army Air Force Medical Center, Beijing, China; ^7^Rehabilitation Department, Haihe Clinical School, Tianjin Medical University, Tianjin, China

**Keywords:** tuberculous meningitis, cerebrospinal fluid examination, lactate level, prognostic factors, retrospective study

## Abstract

**Objective:**

This study aimed to analyze the cerebrospinal fluid (CSF) parameters affecting the outcomes of patients with tuberculous meningitis (TBM).

**Methods:**

This is a multi-center, retrospective, cohort study involving 81 patients who were diagnosed with TBM and treated in Haihe Clinical College of Tianjin Medical University, Tianjin Medical University General Hospital, and General Hospital of Air Force PLA from January 2016 to December 2019. Baseline data, Glasgow Coma Scale (GCS) score, and clinical presentations of all patients were collected at admission. CSF samples were collected at 48 h, 1, 2, and 3 weeks after admission. CSF lactate, adenosine deaminase, chloride, protein, glucose levels and intracranial pressure were measured. After a follow-up of 16.14 ± 3.03 months, all patients were assessed using the modified Rankin Scale (mRS) and divided into good (mRS scores of 0–2 points) and poor outcome groups (mRS scores of 3–6 points). The differences in patients' baseline data, GCS score, clinical presentations, and levels of CSF parameters detected at 48 h, 1, 2, and 3 weeks after admission between two groups were compared. Statistically significant variables were added to the binary logistic regression model to identify the factors impacting the outcomes of patients with TBM. Receiver operating characteristic (ROC) curve was used to assess the predictive ability of the model.

**Results:**

The CSF lactate level exhibited a decreasing trend within 3 weeks of admission in the two groups. For the within-group comparison, statistically significant differences in the lactate level was found in both groups between four different time points. A binary logistic regression model revealed that CSF lactate level at 48 h after admission, age, and GSC score on admission were independently associated with the outcomes of patients with TBM. ROC curve analysis showed that the area under the ROC curve (AUC) was 0.786 for the CSF lactate level (48 h), 0.814 for GCS score, and 0.764 for age.

**Conclusion:**

High CSF lactate level at 48 h after admission is one of the important factors for poor outcomes in patients with TBM.

## Introduction

In 2020, there were 842,000 new cases of tuberculosis (TB) in China, with an incidence rate of 59/100,000. China has the world's second largest TB cases, accounting for 8.5% of the global incidence cases. The number of TB deaths among HIV-negative people was about 30,000, and the TB mortality rate was 2.1 deaths per 100,000 persons (1). Tuberculous meningitis (TBM) is a non-suppurative inflammation of membranes (meninges) surrounding the brain or spinal cord caused by *Mycobacterium tuberculosis*, accounts for about 1–5% of TB cases, approximately one half of patients with TBM die or suffer severe disability (2, 3).

*Mycobacterium tuberculosis* can spread through hematogenous route, produce the formation of granulomas within the ventricles or subarachnoid spaces, and secrete gelatinous exudates (4). The gelatinous exudates can not only block the circulatory path of cerebrospinal fluid (CSF), affect CSF absorption, leading to the formation of hydrocephalus, but also trigger an intense inflammatory response in the middle cerebral artery, vertebrobasilar artery and circle of Willis, which is the main cause of neurological deficits due to cerebral ischemia in patients with TBM (5–7).

The CSF lactate test is a rapid and inexpensive test and good indicator to differentiate bacterial meningitis from aseptic meningitis (8, 9). A prospective cohort study of 176 patients with neurological infections showed CSF lactate had the best diagnostic value, with an area under the curve (AUC) of 0.976, a sensitivity of 96%, and a specificity of 85%. CSF lactate can not only assist physicians in diagnosis but also serve as a predictor to assess patient prognosis (8). CSF lactate is elevated in patients with TBM in the early stage of disease. Therefore, attention should be paid to the changes in CSF lactate and its relationship with patient prognosis. CSF lactate concentration depends on the extent of anaerobic glycolysis in the central nervous system, and has been used to predict the severity of brain parenchymal injury caused by cerebral ischemia and hydrocephalus. The purpose of this study was to analyze the CSF parameters affecting the outcomes of patients with TBM.

## Materials and methods

### Subjects

This is a multicenter, retrospective, cohort study involving 81 patients who were diagnosed for the first time with TBM and treated in Haihe Clinical College of Tianjin Medical University, Tianjin Medical University General Hospital, and General Hospital of Air Force PLA from January 2016 to December 2019. There were 43 males and 38 females, with an average age of 43.04 ± 17.46 years.

The study protocol was approved by the Ethics Committee of our hospital (No. 2022HHWZ-003) and written informed consent was obtained from all patients.

### Inclusion and exclusion criteria

Inclusion criteria were: (1) patients who were aged ≥15 years old; (2) patients who were diagnosed with confirmed TBM according to the TBM diagnostic criteria, i.e., *Mycobacterium tuberculosis* was detected in CSF, or nucleic acid amplification test was positive for *Mycobacterium tuberculosis*; in the absence of evidence of pathogens in the CSF, patients were diagnosed with TBM based on a combination of clinical manifestations, CSF examination, and brain imaging examination (diagnostic score of ≥6), and had a positive result from the T-SPOT*.TB* test (10–12).

Exclusion criteria were: (1) patients who were HIV positive; (2) female patients who were pregnant; (3) patients who were diagnosed with suppurative, cryptococcal, viral, syphilitic, brucella meningitis, brain parasites, malignant tumors, and autoimmune diseases; (4) patients who failed to complete the 1-year follow-up.

### Follow-up and grouping

All patients were followed up for an average of 16.14 ± 3.03 months. The modified Rankin Scale (mRS) was used to assess the outcomes of patients at 1 year after disease onset. All patients were assessed face to face by two senior neurologists who were trained in the use of the mRS. Patients were divided into a good outcome group (mRS scores of 0–2 points) and a poor outcome prognosis group (mRS scores of 3–6 points) (13, 14).

### Sample collection

2 ml of CSF samples were collected by lumbar puncture at 48 h, 1, 2, and 3 weeks after admission, which was then sent to the laboratory of our hospital immediately. Samples were centrifuged at 1,300 *g* for 10 min, and analyzed with a Beckman Coulter AU5800 chemistry analyzer (Brea, CA USA), and reagents from Beckman Coulter (Brea, CA USA). The levels of CSF parameters lactate, adenosine deaminase (ADA), chloride, protein, glucose were detected, and intracranial pressure (ICP) was measured simultaneously.

### Treatment

All patients were treated according to the guidelines for the treatment of TBM, i.e., the duration of intensive-phase treatment for TBM should be > 2 months, the total duration of treatment should be at least 12 months. Patients should receive at least four antitubercular medications during the intensive phase, isoniazid, rifampicin, and pyrazinamide should be used as the preferred antitubercular medications. During consolidation phase, at least two effective antitubercular medications should be given, with isoniazid and rifampicin being used as the preferred medicines (15, 16).

### Statistical analysis

Statistical analysis and graphing were conducted using SPSS 24.0 software, and GraphPad Prism version 9.0. Continuous variables were expressed as mean ± standard deviation (SD), and categorical variables were expressed as number and percentage (%). Continuous variables were compared using the independent or paired *t*-tests, and categorical variables were compared using the Chi-square test or Fisher's exact test. Statistically significant variables were added to the binary logistic regression model. The predictive ability of the model was verified by the receiver operating characteristic (ROC) curve. A *P*-value of < 0.05 was considered statistically significant.

## Results

### The baseline characteristics and clinical manifestations of patients with TBM in the two groups

The average age of patients in the good and poor outcome groups was 54.2 ± 17.6 years old and 38.1 ± 15.1 years old, respectively. Patients in the poor outcome group was significantly older than those in the good outcome group (*P* < 0.001).

In terms of clinical manifestations of TBM, patients in the poor outcome group had a significantly lower Glasgow Coma Scale (GCS) score compared to the good outcome group (*P* < 0.001), and were more likely to present with limb weakness and neck stiffness (*P* < 0.05, Table 1).

**Table 1:** Baseline characteristics and clinical manifestations of patients with TBM in the two groups.

	**Good outcome (*****n*** = **56)**		**Poor outcome (*****n*** = **25)**		***P*-value**
	**Mean/*n***	**SD/%**	**Mean/*n***	**SD/%**	
**Demographic characteristics**					
Age (years)	38.1	15.1	54.2	17.6	< 0.001
Gender (male)	33	58.9%	10	40.0%	0.115
**Symptoms**					
GCS score	14.3	1.3	11.7	2.7	< 0.001
Fever	55	98.2%	24	96.0%	0.525
Headache	51	91.1%	21	84.0%	0.580
Nausea	27	48.2%	14	56.0%	0.517
Limb weakness	38	67.9%	24	96.0%	0.006
Neck rigidity	28	50.0%	20	80.0%	0.011
Cranial nerve palsy	10	17.9%	7	28.0%	0.300

TBM, tuberculous meningitis; GCS, Glasgow Coma Scale.

### Differences in the levels of CSF parameters between the two groups

**Figure 1:**
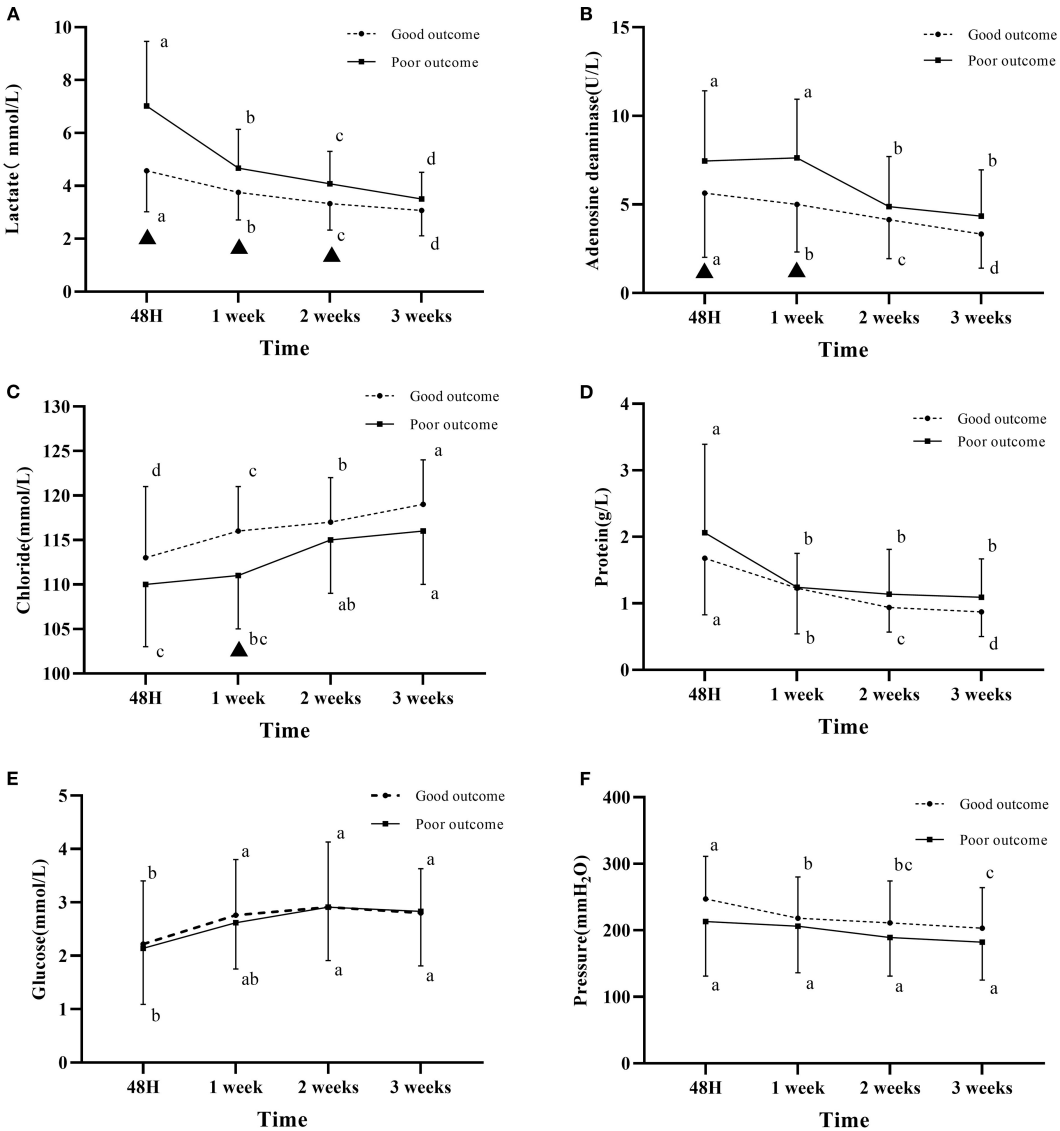
Changes in cerebrospinal fluid parameters over time in the two groups. **(A)** CSF lactate level. **(B)** CSF adenosine deaminase level. **(C)** CSF chloride level. **(D)** CSF protein level. **(E)** CSF glucose. **(F)** The pressure measured by puncture. The letters a, b, c, d indicate within-group comparison between four different time points (values without a common letter are significantly different, values with a common letter are not significantly different); ▴ indicates statistically significant differences between the two groups at the same time point. Good outcome was defined as a modified Rankin scale (mRs) score of 0–2; poor outcome was defined as a mRS score of 3–6.

At 48 h, 1, 2, and 3 weeks after admission, CSF lactate level was significantly higher in the poor outcome group than in the good outcome group (*P* < 0.05, Table 2). The lactate level in the two groups both decreased gradually over time, the decrease was more obvious in the poor outcome group than in the good outcome group within 1 week, whereas the decreasing trend was approximately the same in the two groups after 1 week. For the within-group comparison, statistically significant differences in the lactate level was found in both groups between four different time points (*P* < 0.05, Figure 1).

**Table 2:** Comparison of cerebrospinal fluid parameters between the two groups at four different time points after admission.

	**Good outcome (*****n*** = **56)**		**Poor outcome (*****n*** = **25)**		***P*-value**
	**Mean**	**SD**	**Mean**	**SD**	
**CSF parameters (48 h)**					
Lactate (mmol/L)	4.57	1.55	7.02	2.44	< 0.001
ADA (U/L)	5.64	3.62	7.45	3.97	0.047
Chloride (mmol/L)	112.8	7.5	110.0	6.9	0.121
Protein (g/L)	1.68	0.85	2.06	1.33	0.193
Glucose (mmol/L)	2.22	1.18	2.14	1.05	0.786
Pressure (mmH_2_O)	246.7	64.5	213.4	81.7	0.052
**CSF parameters (1 week)**					
Lactate (mmol/L)	3.75	1.04	4.67	1.47	0.008
ADA (U/L)	5.01	2.69	7.63	3.32	< 0.001
Chloride (mmol/L)	115.8	5.3	111.4	6.2	0.001
Protein (g/L)	1.23	0.69	1.24	0.51	0.914
Glucose (mmol/L)	2.76	1.04	2.62	0.87	0.545
Pressure (mmH_2_O)	217.5	62.3	206.5	70.3	0.482
**CSF parameters (2 weeks)**					
Lactate (mmol/L)	3.33	1.00	4.08	1.22	0.005
ADA (U/L)	4.14	2.19	4.88	2.83	0.200
Chloride (mmol/L)	117.1	5.0	114.8	6.2	0.085
Protein (g/L)	0.94	0.37	1.14	0.67	0.177
Glucose (mmol/L)	2.91	1.22	2.91	1.00	0.985
Pressure (mmH_2_O)	210.6	62.6	189.0	58.0	0.147
**CSF parameters (3 weeks)**					
Lactate (mmol/L)	3.07	0.96	3.50	1.01	0.069
ADA (U/L)	3.33	1.92	4.35	2.61	0.052
Chloride (mmol/L)	118.7	5.0	116.4	5.8	0.075
Protein (g/L)	0.87	0.37	1.09	0.58	0.095
Glucose (mmol/L)	2.80	0.83	2.83	1.02	0.903
Pressure (mmH_2_O)	203.2	60.6	182.4	57.4	0.150

TBM, tuberculous meningitis; CSF, cerebrospinal fluid; ADA, adenosine deaminase.

At 48 h, 1, 2, and 3 weeks after admission, CSF ADA level was higher in the poor outcome group than in the good outcome group. Statistically significant differences in the ADA level between the two groups were found at 48 h and 1 week after admission (*P* < 0.05, Table 2). The ADA level reached its maximum at 1 week of admission, and gradually decreased after 1 week in the poor prognosis group (Figure 1).

At 48 h, 1, 2, and 3 weeks after admission, CSF chloride level was lower in the poor outcome group than in the good outcome group. Statistically significant differences in the chloride level was found between the two groups at 1 week after admission (*P* < 0.05, Table 2). Chloride level gradually increased in the two groups over time (Figure 1).

CSF protein level was similar in the two groups at 1 week after admission, which was higher in the poor outcome group than in the good outcome group at 48 h, 2 and 3 weeks after admission (Table 2). The CSF protein level in the two groups gradually decreased with time. For the within-group comparison, statistically significant differences in the CSF protein level was found in both groups between four different time points (*P* < 0.05, Figure 1).

Within 3 weeks of admission, the pressure measured by lumbar puncture in the two groups was higher than the normal pressure. The pressure gradually decreased over time, which was higher in the good outcome group than that in the poor outcome group (Figure 1).

**Figure 2:**
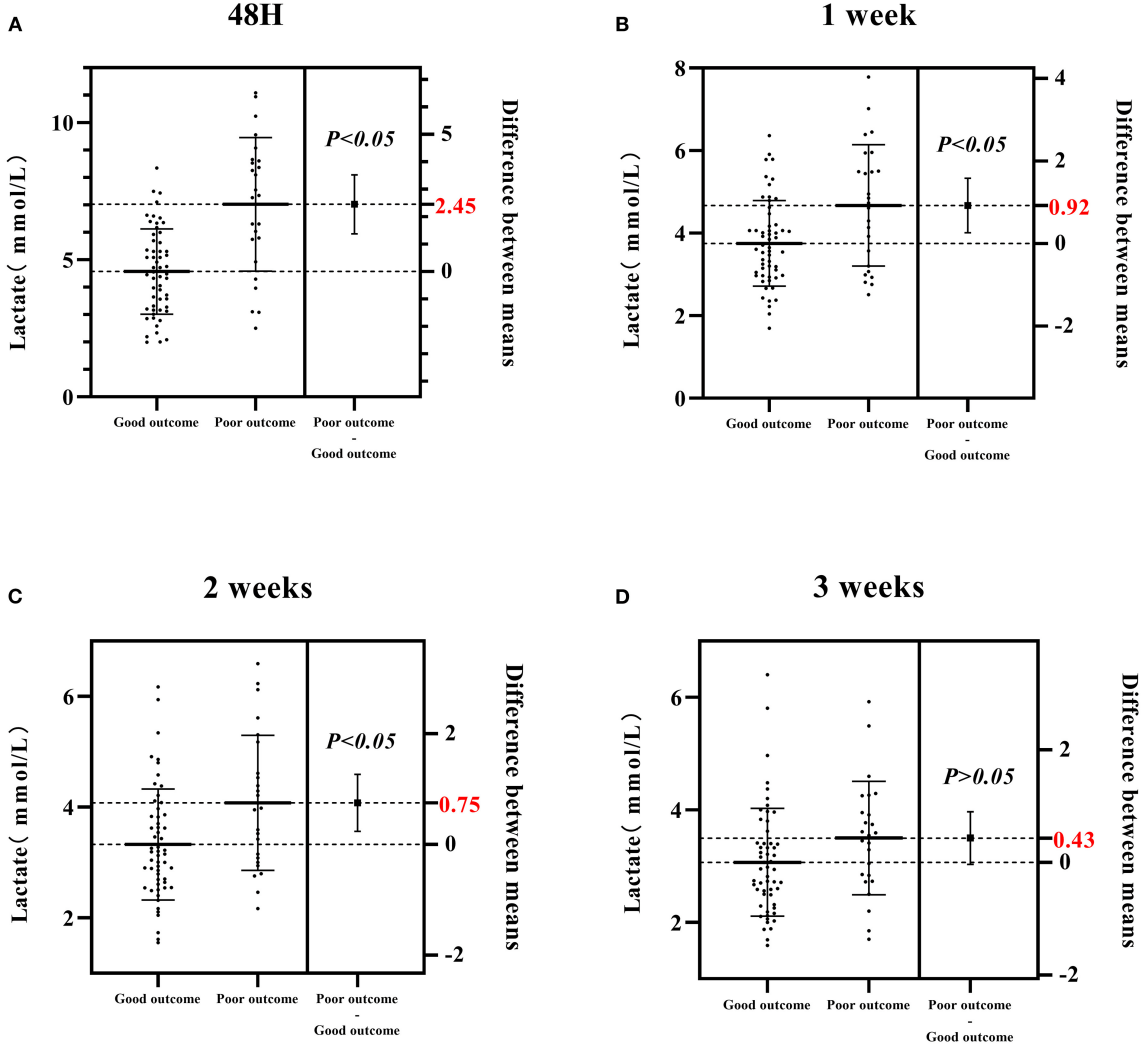
Differences in the CSF lactate level between poor and good outcome groups at four different time points (48 h, 1, 2, 3 weeks) after admission. **(A)** The CSF lactate level in poor and good outcome groups at 48 h after admission. **(B)** The CSF lactate level in poor and good outcome groups at 1 week after admission. **(C)** The CSF lactate level in poor and good outcome groups at 2 weeks after admission. **(D)** The CSF lactate level in poor and good outcome groups at 3 weeks after admission. Good outcome was defined as a modified Rankin scale (mRs) score of 0–2; poor outcome was defined as a mRS score of 3–6.

As shown in Figure 2, differences in the CSF lactate level between poor and good outcome groups gradually decreased over time, with a maximum difference of 2.45 mmol/L at 48 h, and a minimum difference of 0.43 mmol/L at 3 weeks. Statistically significant differences were found between the two groups at 48 h, 1 and 2 weeks (*P* < 0.05), whereas no statistically significant difference was found at 3 weeks (*P* > 0.05, Figure 2).

### Independent factors affecting outcomes in patients with TBM

**Figure 3:**
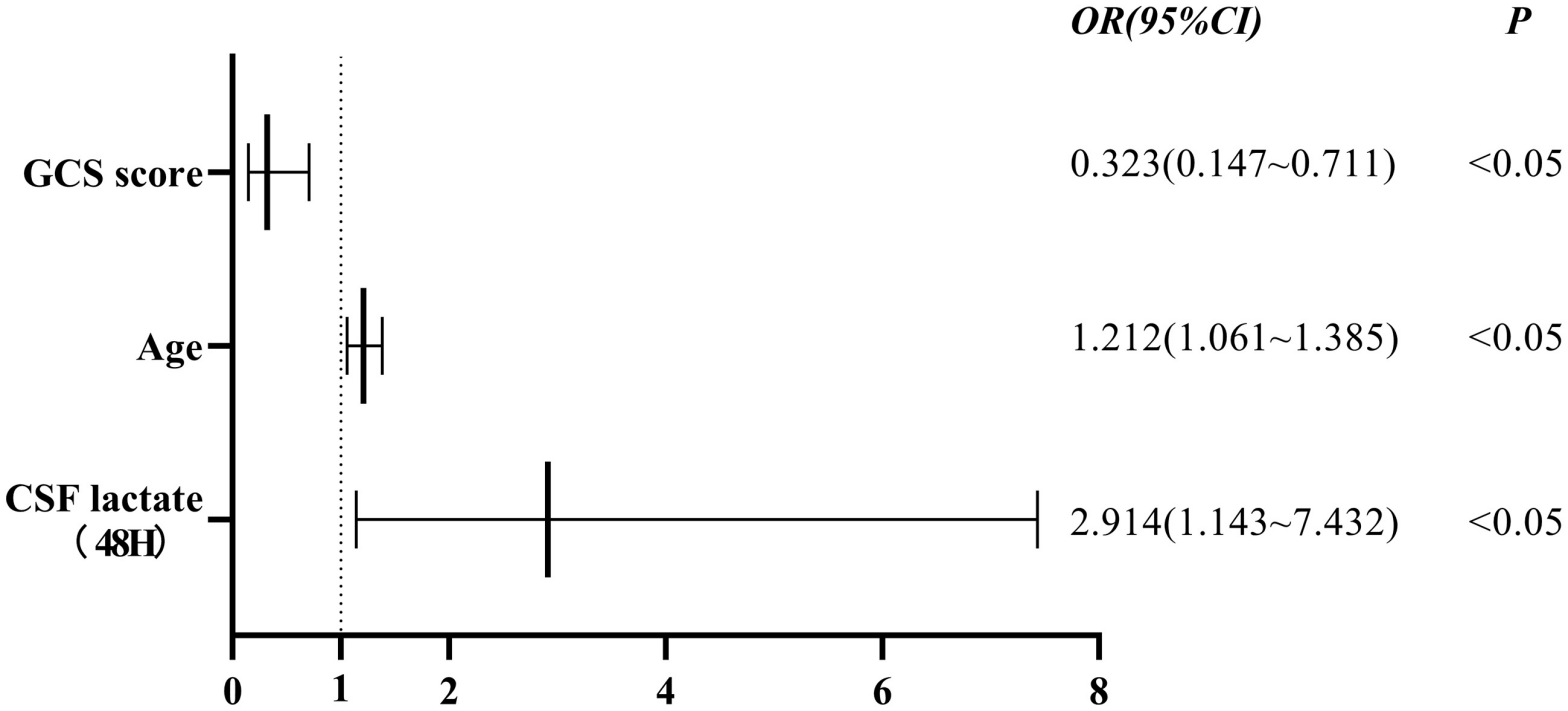
Association between GCS score, age, CSF lactate levels at 48 h after admission and outcomes of patients with TBM. Independent factors associated with the outcomes of TBM patients were GCS score on admission (OR 0.323, 95% CI 0.147–0.711, *P* < 0.05), age (OR 1.212, 95% CI 1.061–1.385, *P* < 0.05), and CSF lactate levels at 48 h after admission (OR 2.914, 95% CI 1.143–7.432, *P* < 0.05). GCS score, Glasgow Coma Scale score; CSF, cerebrospinal fluid; OR, odds ratio; 95% CI, confidence interval.

Statistically significant factors that were shown in Tables 1, 2 were included in the binary logistic regression model. Finally, the results showed that age, GCS score on admission, and CSF lactate levels at 48 h after admission were independent factors associated with the outcomes of TBM patients (Figure 3).

**Figure 4:**
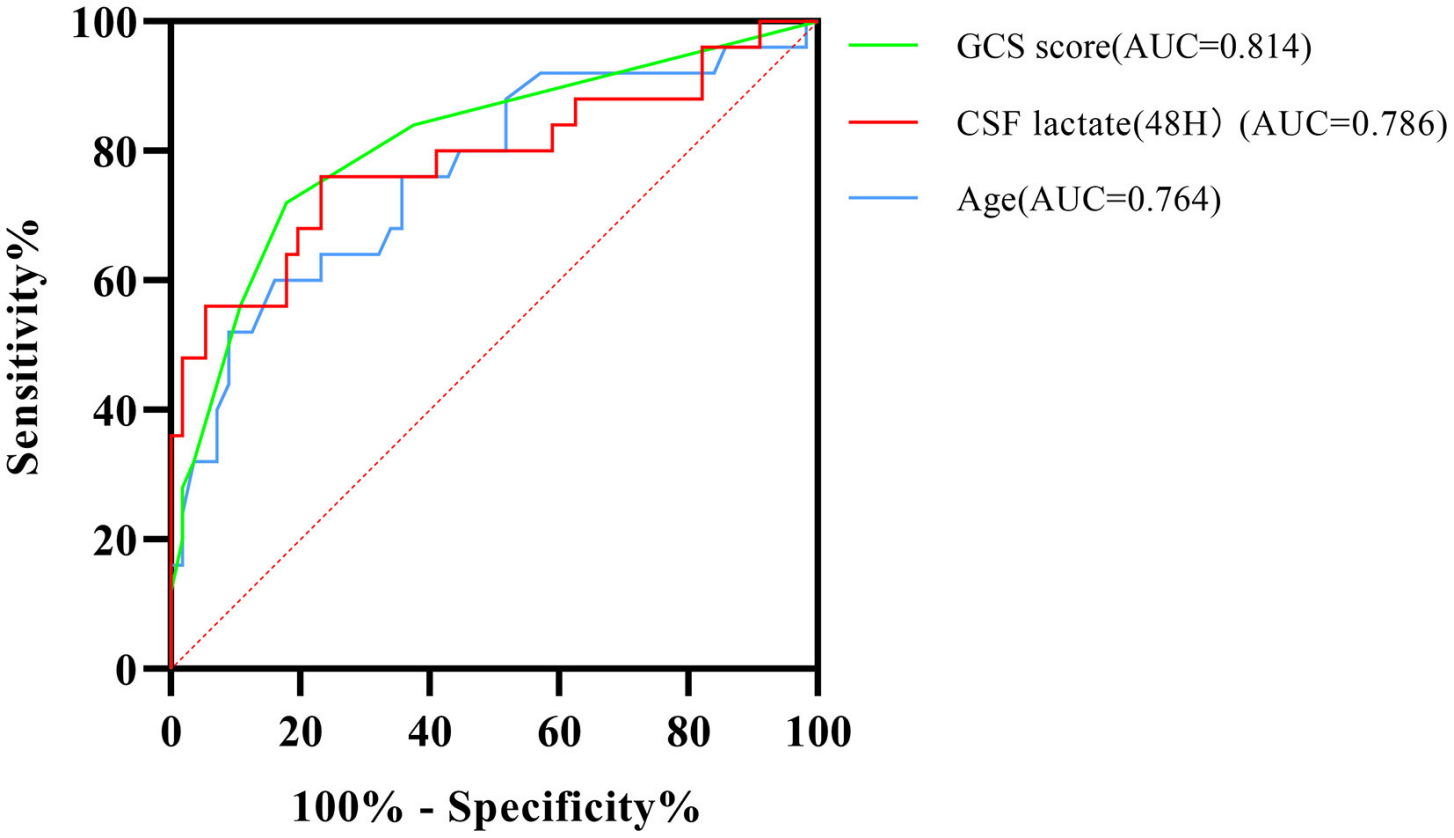
Receiver operating characteristic (ROC) curve analysis shows the accuracy of GCS score, age, and CSF lactate levels at 48 h after admission in predicting prognostic outcomes of patients with TBM. GCS score, Glasgow Coma Scale score; AUC, area under the ROC curve; CSF, cerebrospinal fluid.

ROC curve analysis showed that the area under the ROC curve (AUC) for the CSF lactate level (48 h) was 0.786, with a sensitivity of 76.0%, and a specificity of 76.8%. The AUC value for GCS score was 0.814, with a sensitivity of 72.0%, and a specificity of 82.1%. The AUC value for age was 0.764, with a sensitivity of 60.0%, and a specificity of 83.9%. The ROC curves indicated that the model can accurately predict the outcomes of patients with TBM (Figure 4).

## Discussion

A series of previous studies have demonstrated that TBM had a mortality approaching 20–50% (17–20). A randomized, double-blind, controlled study of adjunctive dexamethasone for the treatment of TBM conducted in Vietnam showed that 50% of patients with TBM died and 14% of survivors presented had severe disability during a 5-year follow-up (21). The neurological sequelae of TBM are mainly caused by hydrocephalus, cerebral ischemia, and tuberculoma (22). About 20–30% of TBM survivors suffer some form of nerve damage, including cranial nerve palsy, ophthalmoplegia, epilepsy, hemiplegia, blindness, deafness, ataxia, psychiatric disorders, and unresponsiveness (17–20).

CSF lactate can be used as a diagnostic tool for many CNS disorders, such as intracranial infections, epileptic seizures, cerebral infarction, and mitochondrial diseases, which is more accurate, and has a higher accuracy than conventional CSF parameters (23, 24). There is no correlation between blood and CSF lactate, because lactate in its ionized state crosses the blood-brain barrier at a very slow rate, which needs to be transported by Monocarboxylate transporters, so CSF lactate is a good metabolic indicator (25–27). Elevated CSF lactate level in patients with TBM is due to increased anaerobic glycolysis caused by cerebral ischemia. Most patients with TBM can experience increased ICP, cerebral blood flow is drastically reduced when the compensation of the ICP reaches its limit. Elevated ICP in patients with TBM is multifactorial, with hydrocephalus, hydrocephalus, and tuberculoma being the most common causes (28, 29). Magnetic resonance spectroscopy demonstrated increased lactate level in the ischemic region in patients with acute cerebral ischemia (30, 31). Microdialysis revealed increased lactate concentration in the extracellular fluid in the ischemic core region, the increase in lactate concentration was low in the peri-ischemic region, whereas increased lactate concentration was not found in the non-ischemic region (32, 33). Lactate can spread from ischemic brain tissues to surrounding normal tissues, which can cause deleterious effects on peripheral nerve cells, and impair cerebral autoregulation, leading to cerebral edema and cerebral ischemia (34). A linear relationship between CSF lactate levels and ischemic edema was found on CT imaging in patients with middle cerebral artery ischemia, lactate level reached its maximum after 3 days of cerebral ischemia. Cerebral edema is more severe when CSF lactate concentration exceeds 4 mmol/L, which is less severe when CSF lactate concentration is below 2.5 mmol/L (35). CSF lactate level is correlated with the time since onset of cerebral ischemia, which reaches a maximal level at 48 h after onset of cerebral ischemia (36). Previous studies have also demonstrated that CSF lactate level was an independent risk factor for the prognosis in patients with TBM, and the CSF lactate concentration was significantly higher in patients with poor prognosis than those with good prognosis (37–39).

Among the prognostic factors of TBM, previous studies have demonstrated that patients' age and GCS score on admission were prognostic factors in patients with TBM (40–44). Results of the present study were consistent with above-mentioned findings, which verified the reliability of the model. The GCS score is a reliable, widely used scale for objectively evaluating the level of consciousness in patients. The GCS is comprised of three components (eye-opening, motor, and verbal responses), has uniform evaluation criteria, and acceptable inter-rater reliability for experienced users. The advantages of GCS score include simplicity, ease of use, without the need for auxiliary diagnostic tools. In the present study, the ROC analysis showed that GCS score on admission had the highest AUC, suggesting that GCS score on admission was the most accurate predictor of outcomes in patients with TBM.

ADA, an enzyme that is widely distributed in tissues and body fluids, has been routinely used to detect *Mycobacterium tuberculosis*. A previous meta-analysis has shown that ADA test is a method for rapid diagnosis of TBM with high sensitivity and specificity, the pooled sensitivity and specificity were 89 and 91%, respectively, indicating that ADA test had adequate accuracy for diagnosing TBM (45). However, the diagnostic value of CSF ADA test remains to be explored. CSF ADA test results can be interpreted in the context of patients' clinical symptoms and laboratory findings, significantly obviously elevated lactate and ADA levels were suggestive of slower clinical recovery and prolonged hospital stay (39, 46).

Elevated CSF protein level is one of the main presentations of TBM. Abnormal protein level in the CSF is associated with the severity of meningeal inflammation and blood-brain barrier dysfunction (47, 48). High protein level in the CSF may cause more formation of basal exudates, leading to cranial nerve involvement (49). CSF protein may serve as a predictor of cranial nerve involvement (50). However, a recent study demonstrated that CSF protein level was usually normal in patients with TBM without clinical presentations such as vomiting and low serum glucose (51).

The results of the present study showed that the ICP assessed by lumbar puncture was lower in the poor outcome group than that in the good outcome group. A previous study showed that there was no correlation between ICP changes and prognosis in patients with TBM (52). The most common cause of elevated ICP in TBM is hydrocephalus (communicating hydrocephalus caused by impaired CSF absorption *via* the arachnoid granulations, or non-communicating hydrocephalus caused by the obstruction of the mesencephalic aqueduct and the fourth ventricle outlets) (53). The pressure measured by lumbar puncture cannot truly reflect ICP, and the use of a transducer placed in the intraventricular, intraparenchymal, and epidural sites is considered the gold standard for ICP monitoring (54). However, this invasive procedure carries the risks of intracranial hemorrhage and infection, and there are no clear guidelines on when ICP monitoring can be performed in patients with TBM and elevated ICP.

A limitation of this study is the small sample size, which can result in wide confidence intervals. Despite these limitations, we believe that early detection of CSF lactate level is important for predicting the outcomes in patients with TBM.

## Conclusion

The present study demonstrated that the levels of CSF parameters at different time points after admission in patients with TBM, especially CSF lactate level at 48 h of admission, are important indicators for predicting the outcomes in patients with TBM. High CSF lactate level at 48 h of admission is an important factor for poor outcomes in patients with TBM.

## Data availability statement

The original contributions presented in the study are included in the article/supplementary material, further inquiries can be directed to the corresponding authors.

## Ethics statement

The studies involving human participants were reviewed and approved by Ethics Committee of Haihe Clinical School, Tianjin Medical University. The patients/participants provided their written informed consent to participate in this study.

## Author contributions

CL, RH, YX, and XH analyzed data and wrote the manuscript. ZC, YLi, XL, HL, HZ, SW, LH, YB, and YLu were responsible for the data collection. YiyW and YiW designed the study and revised the manuscript. All authors have read and approved the final version of the manuscript.

## Funding

This study was supported by Tianjin Science and Technology Project (No. 19ZXYXSY00070) and Tianjin Health Science and Technology Project (No. ZC20145).

## Conflict of interest

The authors declare that the research was conducted in the absence of any commercial or financial relationships that could be construed as a potential conflict of interest.

## Publisher's note

All claims expressed in this article are solely those of the authors and do not necessarily represent those of their affiliated organizations, or those of the publisher, the editors and the reviewers. Any product that may be evaluated in this article, or claim that may be made by its manufacturer, is not guaranteed or endorsed by the publisher.
